# *Daphnia magna* shows reduced infection upon secondary exposure to a pathogen

**DOI:** 10.1098/rsbl.2012.0581

**Published:** 2012-08-08

**Authors:** Seanna J. McTaggart, Philip J. Wilson, Tom J. Little

**Affiliations:** 1 Institute of Evolutionary Biology, University of Edinburgh, Edinburgh EH9 3JT, UK; 2 Centre for Immunity, Infection and Evolution, School of Biological Sciences, Ashworth Laboratories, University of Edinburgh, Edinburgh EH9 3JT, UK

**Keywords:** within-generation immune priming, *Daphnia*, immunological loitering

## Abstract

Previous pathogen exposure is an important predictor of the probability of becoming infected. This is deeply understood for vertebrate hosts, and increasingly so for invertebrate hosts. Here, we test if an initial pathogen exposure changes the infection outcome to a secondary pathogen exposure in the natural host–pathogen system *Daphnia magna* and *Pasteuria ramosa*. Hosts were initially exposed to an infective pathogen strain, a non-infective pathogen strain or a control. The same hosts underwent a second exposure, this time to an infective pathogen strain, either immediately after the initial encounter or 48 h later. We observed that an initial encounter with a pathogen always conferred protection against infection compared with controls.

## 1. Introduction

The threat of infection is a universal challenge, which has led to the evolution of sophisticated defence mechanisms. A defence system that has memory can provide an immense selective advantage, as evidenced by the acquired immune system of jawed vertebrates. However, acquired immunity is a recent and phylogenetically restricted innovation that functions in association with an ancient and universal system, innate immunity. It was long thought that innate immune responses do not change after repeated exposures. However, experiments in invertebrates, which possess only the innate immune system, demonstrate a phenotypic response called immune priming, where previous pathogen exposure results in increased host protection to subsequent pathogen exposures. The term immune ‘priming’ is used to distinguish the effect from the functionally similar, but mechanistically different, response of the acquired immune system. The prophylactic effect of immune priming has been found in a wide-range of invertebrate taxa and can occur within an individual's lifetime [1–3] or across generations [3–5]. Although the mechanistic underpinnings of immune priming are not well understood (but see [6,7]), what is clear is that the innate immune response is more efficacious after a preliminary encounter [8].

Here we used the crustacean *Daphnia magna* and its naturally infecting bacterial pathogen *Pasteuria ramosa* to study immune priming. The essential design feature of any such study is dual exposure, where hosts are challenged (the primary exposure), and later challenged again (the secondary exposure). Previous studies on this host–pathogen system demonstrated transgenerational immune priming, where mothers exposed to particular pathogen strains gave birth to offspring with enhanced resistance when exposed to the same pathogen strain [5]. The present study focused on effects within a generation. We undertook a variety of dual exposure treatments by varying the parasite strain used in the primary exposure, and/or the time between the primary and secondary exposures.

## 2. Material and methods

We used two *Daphnia* clones: GG1 from a pond near Gaarzerfeld, Germany and FS24 from Kaimes Pond, Scotland. *Daphnia* ingest *P. ramosa* spores released from decaying, infected *Daphnia* cadavers, and infection is associated with a severe reduction in host fecundity. The two *P. ramosa* isolates used in this study (Sp1 and Sp24) originated from the same ponds as the host clones (Sp1, Gaazerfeld; Sp24, Kaimes Pond) and are highly infective to the genotype from their pond of origin, but not to the host genotype from the other pond ([9]; T. Little 2012, unpublished data).

Suspensions of crushed, infected *Daphnia* containing 50 000 spores ml^−1^ were prepared for both parasite strains on the day that exposures took place. The control solution consisted of uninfected crushed *Daphnia*. Exposures took place in 1.5 ml, 24-well plates (Corning, Costar), where one *Daphnia* was placed in a well with spores or control solution for 4 h. When out of the infection chambers, *Daphnia* were housed in 60 ml jars in incubators (20°C, 12 L : 12 D cycle), fed 5 × 10^6^ algal cells (*Chlorella* sp.) per *Daphnia* per day and the medium changed every other day.

**Figure 1.**
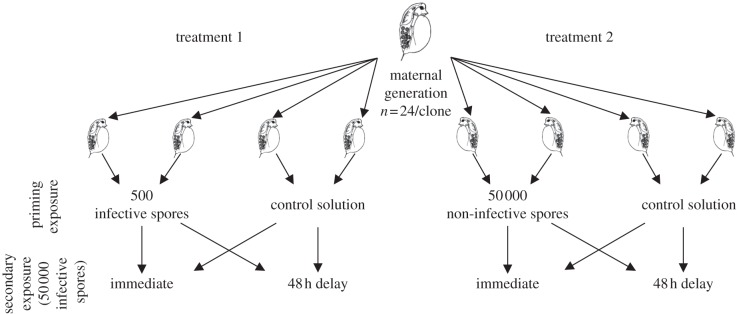
Experimental design. For each of *Daphnia* genotype GG4 and FS24, 24 mothers each supplied eight, 5 day old neonates, which were randomly distributed to one of eight experimental treatments. In treatment 1, the initial exposure consisted of 500 infective *Pasteuria* spores (i.e. Sp1 for GG1 *Daphnia* clones, and Sp24 for FS24 *Daphnia* clones). In treatment 2, the initial exposure consisted of 50 000 non-infective *Pasteuria* spores (i.e. Sp1 for *Daphnia* FS24 clones, and Sp24 for *Daphnia* GG1 clones). Across both treatments the secondary exposure always consisted of 50 000 infective *Pasteuria* spores, and occurred immediately after the initial exposure, or 48 h later. All individuals were monitored for infection for 28 days.

Prior to experimentation, *Daphnia* were kept for three generations under these same conditions. Experimental animals were taken from the third clutch of babies born to the third generation of maternal *Daphnia*. We used a split-clutch design such that eight neonates were taken from each of 24 *Daphnia* and assigned to experimental treatments as follows and described in figure 1.

In our experiment, we sought to use an initial infection protocol that would not lead to infection, but was a genuine exposure to a live pathogen with the potential to stimulate a defence response (figure 1). The first treatment involved an initial exposure of 500 infective spores, which is a dose unlikely to cause infection. Thus, GG1 was exposed to Sp1, and FS24 was exposed to Sp24. The second treatment was to expose *Daphnia* to an initial exposure of 50 000 spores of a non-infective parasite strain. Thus, GG1 was exposed to Sp24, and FS24 was exposed to Sp1. Each treatment had independent sets of controls, which were pooled in the statistical analysis.

In both treatments, the secondary exposure consisted of exposing both hosts to 50 000 infective spores or to a control solution (figure 1). However, we varied the timing of the secondary exposure, such that it occurred either immediately after the completion of the 4 h primary exposure, or 48 h later (figure 1). In sum, for each of the two *Daphnia* clones, there were eight treatments with 24 replicates for a total of 384 *Daphnia. Daphnia* were monitored for infection by eye for 28 days after the initial exposure. All data were analysed in JMP v. 8.0. We modelled the binary response variable infection status using a binomial distribution with a logit link function. We included host genotype, primary infection and timing of secondary infection as main effects and also tested for all interactions.

## 3. Results

**Figure 2.**
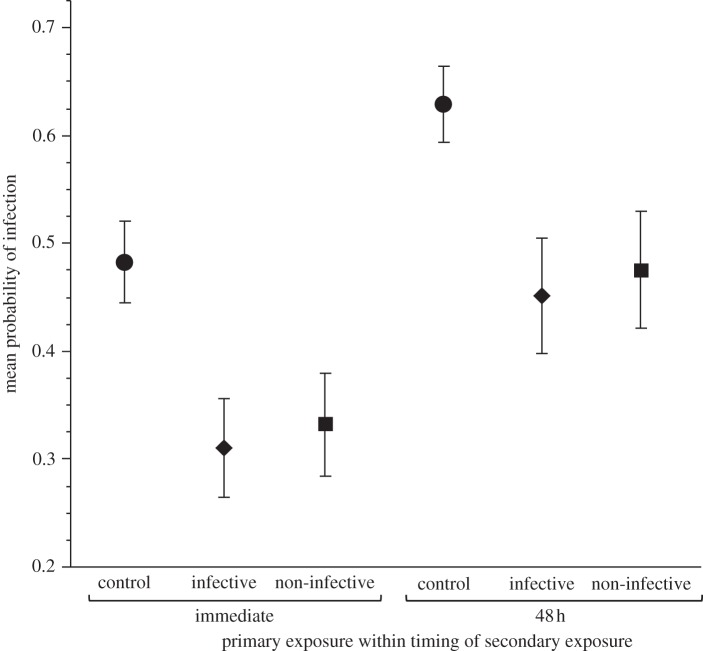
Mean probability of infection in three primary exposure treatments (exposed to control solution (pooled from both experiments), an infective pathogen strain, a non-infective pathogen strain), after a secondary exposure to an infective pathogen strain that occurred immediately following the primary exposure, or 48 h later. Error bars indicate 95% CIs.

Clone, type of primary exposure and the timing of the secondary exposure all had a significant effect on the infection status of the host (table 1). None of the interaction terms were significant. Since the interaction of host clone and treatment was not significant, we calculated and present the mean probability and 95% CIs of infection status over both genotypes as determined by the statistical model (figure 2).

**Table 1.** Summary of analysis of infection status in primed and control *Daphnia*. The effects tested were *Daphnia* genotype (GG1 and FS24), primary exposure (infective spores, non-infective spores, control), and timing of secondary exposure (immediate, 48 h post primary infection). d.f. = Degrees of freedom.

infection status	d.f.	χ^2^	*p*-value
clone	1	38.97	<0.001
primary exposure	2	10.53	0.0052
timing of 2nd exposure	1	7.85	0.0051
clone × primary exposure	2	2.67	0.2632
clone × timing of 2nd exposure	1	1.40	0.2371
clone × primary × timing of 2nd exposure	2	0.90	0.6360

## 4. Discussion

We observed that infection rates were reduced by earlier exposure to parasite spores. This was true irrespective of the type of primary pathogen exposure, or of the timing of the secondary exposure. This demonstrates that *D. magna* undergo a robust, within-generation priming response to their natural pathogen, *P. ramosa*.

The infection process in this system is classically divided into two distinct components: (i) parasite entry across host barriers, i.e. the gut, and (ii) within-host parasite growth, and the consequent host physiological and immune *responses* to the parasites [10–12]. Duneau *et al.* [11] found that, 24 h post-exposure, infective (i.e. genetically compatible) pathogen spores attached to the *D. magna* oesophagus, but non-infective (i.e. genetically incompatible) spores did not attach to an observable degree. Thus, parasite entry in this system appears to be at the oesophagus, and may be mediated by constitutively expressed proteins in the host. Related to this, another study observed that *D. magna* genotypes launch a systemic cellular response only when they are exposed to infective *P. ramosa* spores, which suggests that non-infective spores do not cross barrier defences [12,13].

Thus, it is clear that resistance is partly determined at the point of parasite entry, that particular host–parasite combinations are either compatible or not (a pattern termed genetic specificity), and that compatibility is a strong predictor of the strength of the cellular immune response. And yet, within infective combinations, the probability of infection is dependent on a number of factors, including host genotype, current environmental conditions and maternal environmental conditions [10,14,15]. Our results from treatment 1 add another factor; in particular, primary exposure with genetically compatible parasite strains resulted in decreased infection upon secondary exposure. Further, and perhaps surprisingly, our results from treatment 2 suggest that genetically incompatible, non-infective *P. ramosa* spores also stimulate a protective response, and thus must be detected by the host, despite the evidence that indicates that these spores do not attach and penetrate the host. We surmise that *P. ramosa* are either detectable as they travel through the gut lumen without attachment, or that they are bound and released before 24 h post exposure. Since we found that exposure to non-infective spores results in decreased infection after secondary exposure, but rarely results in a cellular response [12,13], phagocytes in the haemolymph are unlikely to be the primary mechanism behind immune priming.

Our results differ from previous immune priming studies in two respects. First, the fact that the observed immune priming response is equivalent when the primary and secondary exposures were executed with the same (treatment 1) or with different pathogen strains (treatment 2) indicates that priming is not specific in this context. In contrast, immune priming has been shown to be specific in several taxa [2,6,7,16]. Secondly, the inference that the cellular response may not play a role in the immune priming response differs from results of experiments in *Drosophila*, which demonstrate that phagocytosis is a necessary component of immune priming [7]. Taken together, these observations suggest that mechanisms of immune priming may not be homologous across invertebrate taxa.

Gene expression profiling in *Drosophila* has shown that effector molecules are largely cleared from the haemolymph 48 h after pathogen exposure, while in *Daphnia*, phagocytes induced by infective *P. ramosa* spores are cleared from the haemolymph by 24 h [12]. If priming is solely dependent on the loitering of these immune molecules, then one would predict that the effect would decay over time in proportion with the clearing of the molecules from the haemolymph. We chose the 48 time point for the purpose of testing this hypothesis. The number of individuals infected was higher at 48 h than immediately after the initial pathogen exposure. However, the *relative* difference between controls and ‘primed’ individuals does not appear to change with timing of the secondary exposure. Since we do not observe decay in the priming response, it is unlikely that priming is solely driven by a simple immunological loitering effect.

Indeed, mammalian innate immune cells undergo profound changes after exposure to a pathogen. For example, natural killer (NK) cells produce long-lived cell populations after exposure to a pathogen, which clonally expand upon secondary challenge and mediate a response that results in specific, increased protection compared with naive NK cells [17,18]. The fact that an innate immune cell type exhibits traits that were previously thought to be unique to adaptive immune cells suggests a mechanistic framework for understanding the phenomenon of innate immune priming.
